# *N*‐carbamoylglutamate‐responsive carbamoyl phosphate synthetase 1 (CPS1) deficiency: A patient with a novel CPS1 mutation and an experimental study on the mutation's effects

**DOI:** 10.1002/jmd2.12034

**Published:** 2019-05-02

**Authors:** Sufin Yap, Nadine Gougeard, Anthony R. Hart, Belén Barcelona, Vicente Rubio

**Affiliations:** ^1^ Department of Inherited Metabolic Disorders Sheffield Children's Hospital Sheffield UK; ^2^ Structural Enzymopathology Unit Instituto de Biomedicina de Valencia of the CSIC (IBV‐CSIC) Valencia Spain; ^3^ Group 739, Centro de Investigación Biomédica en Red para Enfermedades Raras (CIBERER‐ISCIII) Madrid Spain; ^4^ Department of Neurology Sheffield Children's Hospital Sheffield UK

**Keywords:** carbamylglutamate, carglumic acid, carglumic acid test trial, hyperammonemia, novel treatments, urea cycle disorders

## Abstract

*N*‐carbamoyl‐l‐glutamate (NCG), the *N*‐acetyl‐l‐glutamate analogue used to treat *N*‐acetylglutamate synthase deficiency, has been proposed as potential therapy of carbamoyl phosphate synthetase 1 deficiency (CPS1D). Previous findings in five CPS1D patients suggest that NCG‐responsiveness could be mutation‐specific. We report on a patient with CPS1D, homozygous for the novel p.(Pro1211Arg) CPS1 mutation, who presented at 9 days of life with hyperammonemic coma which was successfully treated with emergency measures. He remained metabolically stable on merely oral NCG, arginine, and modest protein restriction. Ammonia scavengers were only added after poor dietary compliance following solid food intake at age 1 year. The patient received a liver transplantation at 3.9 years of age, having normal cognitive, motor, and quality of life scores despite repeated but successfully treated episodes of hyperammonemia. Studies using recombinantly produced mutant CPS1 confirmed the partial nature of the CPS1D triggered by the p.(Pro1211Arg) mutation. This mutation decreased the solubility and yield of CPS1 as expected for increased tendency to misfold, and reduced the thermal stability, maximum specific activity (*V*_max_; ~2‐fold reduction), and apparent affinity (~5‐fold reduction) for ATP of the purified enzyme. By increasing the saturation of the NAG site in vivo, NCG could stabilize CPS1 and minimize the decrease in the effective affinity of the enzyme for ATP. These observations, together with prior experience, support the ascertainment of clinical responsiveness to NCG in CPS1 deficient patients, particularly when decreased stability or lowered affinity for NAG of the mutant enzyme are suspected or proven.

**Synopsis**

The novel p.(Pro1211Arg) CPS1 mutation causes partial but life‐threatening CPS1 deficiency that may be alleviated by administration of *N*‐carbamoylglutamate, a drug that should be clinically tested for responsiveness in CPS1 deficient patients.

## 1 INTRODUCTION

Carbamoyl phosphate synthetase 1 deficiency (CPS1D), a rare autosomal recessive inborn error of the urea cycle, is due to loss‐of‐function *CPS1* gene mutations.^1^ In CPS1D, hyperammonemic crises compromise life and cognitive status, requiring prompt treatment by protein withdrawal, prevention of catabolism, ammonia scavengers, arginine or citrulline, and hemofiltration.^2^ Maintenance treatment aims at preventing decompensations by protein restriction, oral ammonia scavengers plus arginine or citrulline, and eventually, curative liver transplantation.^2^

Carbamoyl phosphate synthetase 1 deficiency prognosis remains poor,^3^ highlighting the need for additional therapies.^4^ One such therapy could be *N*‐carbamoyl‐l‐glutamate (NCG) use. This deacylase‐resistant analogue of *N*‐acetyl‐l‐glutamate (NAG), the essential activator of CPS1,^5^ is used in primary NAG synthase (NAGS) deficiency^2^ and the hyperammonemia of organic acidemias.^6^ It could be valuable in partial CPS1D if NAG fails to saturate CPS1 in vivo, which might be common in urea cycle disorders if liver glutamate levels (needed for NAG synthesis; NAGS has a high *K*_m_ for glutamate^7^) are low due to therapeutic protein restriction and to increased glutamate conversion to glutamine. Forced NAG site saturation by NCG might not only maximize CPS1 activation but could also protect CPS1 from irreversible inactivation because NAG binding in conjunction with MgATP protects CPS1 against thermal and proteolytic inactivation.^8^ Thus, NCG could act as a chemical chaperone, being valuable for CPS1 destabilization‐causing mutations.

Although advocated for many years,^9^, ^10^ there are few reports on the use of NCG in CPS1D.^11^, ^12^ Results in five late‐onset CPS1D patients were encouraging,^12^ although a paradoxical negative biochemical effect in one patient led to propose individual CPS1 mutations‐based counselling.^13^ We report here a patient with genetically characterized partial but severe CPS1D due to homozygosity for the novel c.3632C>G, p.(Pro1211Arg) mutation that was controlled for a long period with merely oral NCG, arginine, and moderate protein restriction. Our case, with normal neurodevelopmental outcome, illustrates how a CPS1 mutation having no apparent direct effects on NAG binding to the enzyme could be sensitive to NCG administration, supporting the convenience of clinically testing the efficacy of NCG in every patient with partial CPS1D.

## 2 PATIENTS AND METHODS

### 2.1 Patient and CPS1 diagnostic procedures

All the interventions of this retrospective, observational review of medical records were intended for diagnosis and treatment and thus did not require ethical committee review. Written informed consent was obtained from the patient's mother for clinical and laboratory procedures and for data publication.

Carbamoyl phosphate synthetase 1 deficiency was suggested by the clinical and laboratory data (see Section 3). It was confirmed by automated Sanger DNA sequencing of all the polymerase chain reaction (PCR)‐amplified exons and exon/intron boundaries of the *CPS1* gene, using blood DNA as template and appropriate oligonucleotide pairs (sequences will be provided upon request). Reference genomic and mRNA sequences are those with respective GenBank entries NG_008285.1 and NM_001875.4 (https://www.ncbi.nlm.nih.gov/nucleotide/). Nucleotide numbering of the mRNA starts at the A of the ATG translation initiation codon (codon 1). The translated protein sequence of human CPS1 corresponds to entry P31327 of UniProtKB (https://www.uniprot.org/uniprot/).

### 2.2 Recombinant production of wild type and mutant CPS1

Human mature CPS1 was produced recombinantly in a baculovirus/insect cell system.^8^ The mutation c.3632C>G causing the predicted p.(Pro1211Arg) substitution was introduced into the pFastBac‐CPS1 plasmid as reported for other mutations^8^ using the respective forward and reverse oligonucleotides, 5′CTCTGATGCTGCGCACACAAACC3′ and 5′ GTTTGTGTGCGCAGCATCAGAGTG3′ (the changed nucleotide is underlined), corroborating the correctness of the change and the lack of unwanted changes by automated Sanger sequencing. Wild type and mutant CPS1 were purified from baculovirus‐infected insect cell extracts by Ni^2+^‐affinity chromatography.^8^ Purity of the final soluble CPS1 preparations was assessed by polyacrylamide gel electrophoresis in the presence of sodium dodecyl sulphate (SDS‐PAGE; 8% polyacrylamide gels^14^), with densitometric quantification of Coomassie‐stained band intensities (Multi Gauge software; Fujifilm). Total protein was determined according to Bradford^15^ using bovine serum albumin as standard.

### 2.3 Enzyme activity assays

Carbamoyl phosphate synthetase 1 activity was determined colorimetrically as citrulline production^16^ after 10‐minutes incubation at 37°C in a solution of 50 mM glycylglycine pH 7.4, 70 mM KCl, 1 mM dithiothreitol, 35 mM NH_4_Cl, 50 mM KHCO_3_, 5 mM adenosine‐5‐triphosphate (ATP), 25 mM MgSO_4_, 10 mM NAG, 5 mM L‐ornithine HCl, and 4 U/mL of pure *Enterococcus faecalis* ornithine transcarbamylase (produced recombinantly in our laboratory). To study substrate kinetics and NAG activation, the concentration of each substrate or of NAG was varied individually while other assay components were kept fixed at the concentrations used in the standard reaction assay, with MgSO_4_ being always in 20 mM excess over ATP. Kinetic data were fitted to hyperbolae (GraphPad Prism; GraphPad Software, San Diego, California).

For monitoring the thermal stability of CPS1, a solution of 0.5 mg/mL of this enzyme (wild type or mutant) in 50 mM glycylglycine pH 7.4, 20 mM imidazole, 0.5 M NaCl, 2 mM dithiotreitol (DTT), and 10% glycerol was heated 15 minutes at the indicated temperature, then chilled to 0°C, followed by immediate CPS1 activity determination at 37°C as described above.

### 2.4 In silico studies

Amino acid conservation was determined by sequence alignment with Clustal (https://www.ebi.ac.uk/Tools/msa/clustalo)^17^ of either CPS1, CPSIII (the piscine form; it uses glutamine and it is partially activated by NAG) or other CPSs from 14, 6, and 24 species, respectively. The on‐line servers PolyPhen‐2 (http://genetics.bwh.harvard.edu/pph2; HumVar‐trained dataset),^18^ MutPred2 (http://mutpred2.mutdb.org),^19^ and Mutation Taster2 (http://www.mutationtaster.org)^20^ were used for prediction of the functional impact of the p.(Pro1211Arg) missense mutation. PolyPhen‐2 grades the probability of a damaging effect as *probably damaging*, *possibly damaging*, and *benign*, with the highest probability score being 1. The score given by MutPred2 is the probability that a given amino acid change is deleterious/disease associated. Pymol (DeLano Scientific; http://www.pymol.org) was used for visual structural analysis and for depicting the CPS1 structure.

## 3 RESULTS

### 3.1 Patient data

**Figure 1:**
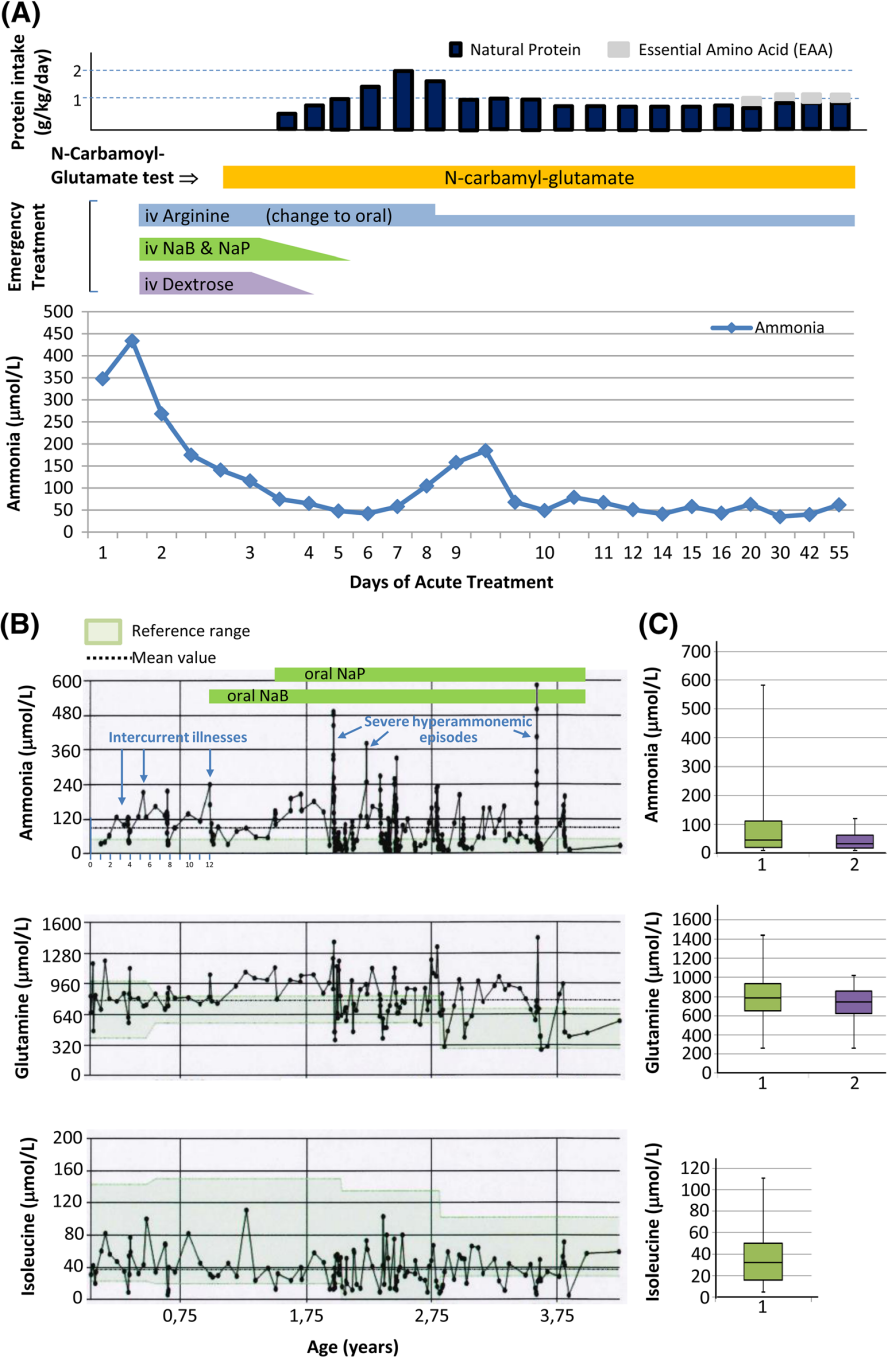
Patient data. NaB and NaP stand for Na benzoate and Na phenylbutyrate, respectively. A, Protein intake (top), administration of drugs (middle), and ammonia levels (bottom) until metabolic stabilization during the first hyperammonemic crisis, plotted vs days from start of intervention (day 1 corresponds to day 9 of life). B, Lifelong levels of plasma ammonia (top), glutamine (middle), and isoleucine (bottom), excluding the initial period of presentation and stabilization. The colored horizontal bands denote the addition of ammonia scavengers, as indicated, to the treatment regime of the patient. Arrows indicate febrile events associated with increased ammonia levels and very severe events that required hemofiltration, as specified. C, Corresponding boxplots for the lifelong ammonia, glutamine, and isoleucine levels including (1) or excluding (2) levels during periods of decompensation. EAA, essential amino acid

A patient born normal at full term and discharged at 2 days was admitted on day 9 of life with hyperammonemic coma (plasma ammonia of 348, escalating to 434 μmol/L; ref: for first month of life <100 μmol/L, and afterward, <50 μmol/L; Figure 1A, bottom panel). Application of emergency measures (protein withdrawal, intravenous 10% dextrose, sodium benzoate, sodium phenylbutyrate, and arginine) as recommended for undiagnosed hyperammonemia in www.bimdg.org.uk/store/guidelines/Hyperammonaemiaand_manage_2016_415469_09092016.pdf, led to recovery from coma in 18 hours, with ammonia levels decreasing to <150 μmol/L (Figure 1A, bottom panel). Plasma citrulline and glutamine were, respectively, undetectable (ref: 5‐36) and in the high‐normal range (830 μmol/L; ref 392‐988). Two male siblings born abroad had died neonatally with purported sepsis. The patient presented with no acidosis, urinary ketones, or orotic acid (determined by GC‐MS). In view of all of this, CPS1D or NAGS deficiency (NAGSD) were suspected,^2^ leading to administration of NCG^21^ by nasogastric tube (Carbaglu 200 mg bolus followed by 200 mg/day [63 mg/kg] in four doses; Figure 1A, middle panel). Ammonia decreased to normal (Figure 1, bottom panel) with the stabilization of the patient from day 14 of life solely on oral NCG (63 mg/kg/day, in four doses) and arginine (300 mg/kg/day), while natural protein in his formula was increased gradually, although it had to be restricted to 0.8 g/kg/day to curb increasing ammonia levels (Figure 1A). Later on, a further 0.4 g/kg/day of essential amino acids (EAAs) were added (Figure 1A, top panel). The ammonia levels remained normal for about 2 months. Later on, even during intercurrent infections, ammonia levels did not exceed 130 μmol/L. Meanwhile CPS1D was confirmed genetically (see below). Metabolic control deteriorated after introduction of solid foods at about 6 months of age, suggesting poorer compliance with protein restriction, with ammonia values increasing to about 240 μmol/L during intercurrent illness (Figure 1B, top panel, marked with arrows), leading to the addition at 1 year of age of oral sodium benzoate (200 mg/kg/day), followed half a year later by sodium phenylbutyrate (200 mg/kg/day; Figure 1B, top panel). During hospital admissions, when good compliance with protein restriction was achieved through nasogastric feeding, his biochemical control was easily achieved solely on oral NCG (about 60 mg/kg/day to the nearest 50 mg Carbaglu tablet), arginine, and the above indicated moderate protein restriction, without ammonia scavengers.

Despite suffering three severe episodes of metabolic decompensation, secondary to infections, that required hemofiltration (Figure 1B, marked on top panel), his neurodevelopmental status, assessed at age 3 years,^22^ was in the low‐normal range for the Bayley scales (16th and 12th centiles of the composite cognitive and motor scores, respectively; fine and gross motor scaled scores, 8 and 6, respectively; communication scores not assessed because English was not the patient's first language). Quality of life score was 84.7 (PedsQL version 4.0, for 2‐4 years).^23^ At age 3.9 years, the patient underwent a successful liver transplantation. Growth parameters at age of transplantation (weight, length, and head circumference in the 25‐50th, 50th, and 25‐50th percentiles, respectively) and lifetime ammonia, glutamine, and isoleucine levels (Figure 1B,C) revealed reasonably good overall biochemical control and protein intake.

### 3.2 Molecular diagnosis and pathogenicity predictions

**Figure 2:**
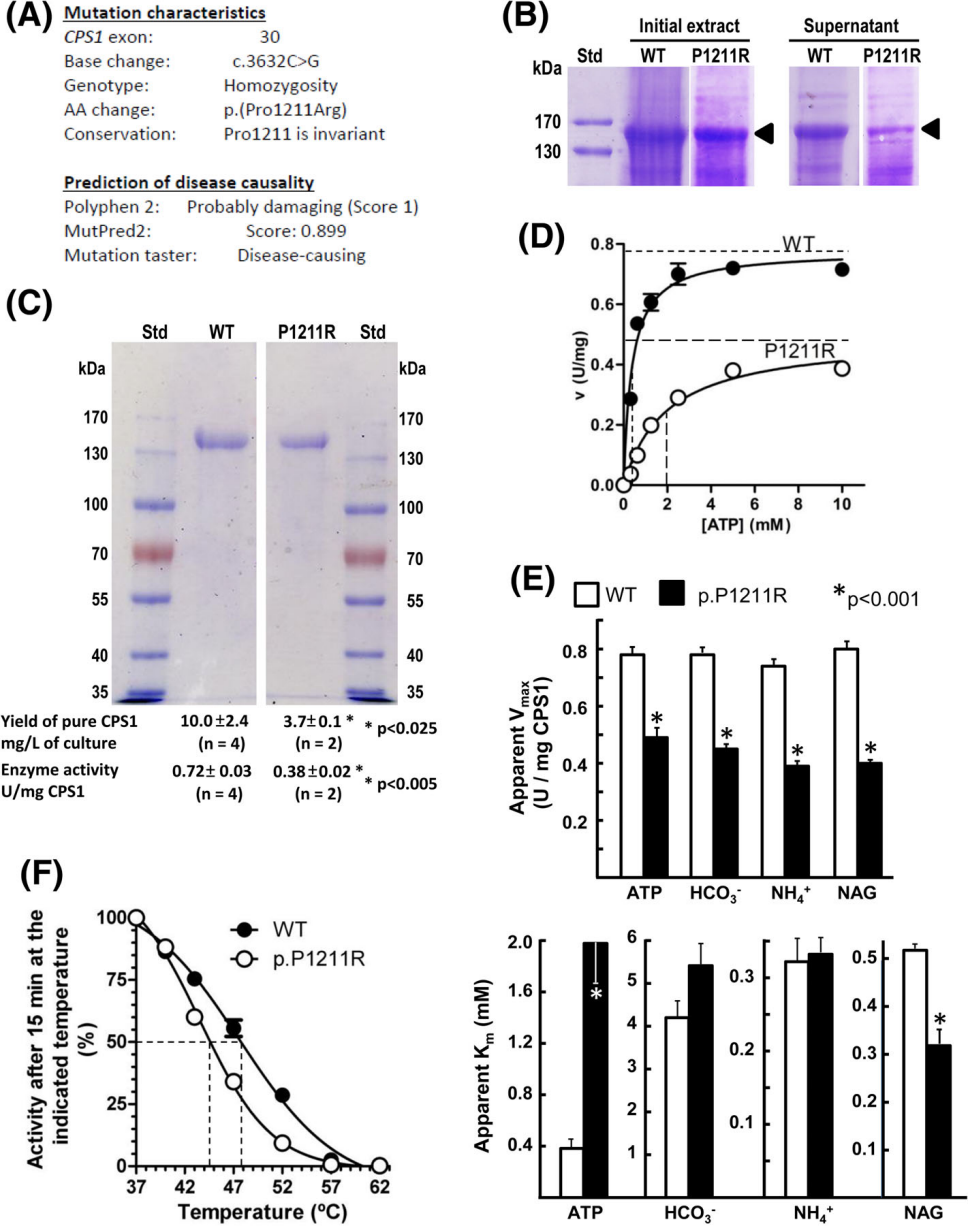
The p.(Pro1211Arg) mutation. A, Summary of the characteristics and in silico predictions for the mutation identified in our patient. (B) and (C) SDS‐PAGE of prestained protein standards (St) of the indicated masses (given on the side in kDa) and of wild‐type (WT) and p.(Pro1211Arg) (P1211R) CPS1 mutant of (B) total crude insect cell extracts and of the postcentrifugal supernatant, as indicated, to show the decreased solubility of the mutant form relative to WT CPS1 (CPS1 band marked with arrowpoint); or (C) after enzyme purification, with yields and enzyme activities of the purified proteins given below the tracks. Significant differences (Student *t* test) are given with an asterisk. D, Illustration of substrate kinetics for ATP for the wild type and the mutant form. Horizontal broken lines mark apparent *V*_max_ values and vertical broken lines mark apparent *K*_m_^ATP^. E, Summary of *V*_max_ (upper panel) and *K*_m_ (lower panel) values for all three substrates. The asterisks denote significant differences for the indicated *P* value. F, Thermal inactivation experiments (for details see Section 2). Activities are expressed as a percentage of the activities observed after incubation at 37°C (at this temperature none of the two protein forms exhibited substantial activity losses). The broken vertical lines mark the temperatures of mid‐inactivation. CPS1, carbamoyl phosphate synthetase 1; NAG, *N*‐acetyl‐l‐glutamate

No mutation was found in the seven exons and the flanking intronic sequences of the *NAGS* gene. In contrast, both alleles of the *CPS1* gene presented in homozygosity (Figure 2A) the nonsynonymous single‐nucleotide change c.3632C>G affecting base 74 of exon 30 of the *CPS1* gene, corresponding to the predicted amino acid substitution p.(Pro1211Arg). This base change was not found in the EXAC (http://exac.broadinstitute.org/transcript/ENST00000233072), 1000 Genomes (https://www.ncbi.nlm.nih.gov/variation/tools/1000genomes/), dbSNP (https://www.ncbi.nlm.nih.gov/projects/SNP/snp_ref.cgi?geneId=1373), or specific CPS1D LOVD databases (https://databases.lovd.nl/shared/genes/CPS1) and therefore it is a novel mutation. The proline that is mutated is invariant among all types of CPS. Three pathogenicity prediction servers (Figure 2A) were coincident in considering the mutation as disease causing. Therefore, this mutation is highly likely to be the cause of CPS1D and of the resulting hyperammonemia observed in this patient.

### 3.3 Functional studies with isolated recombinant CPS1

We corroborated the disease‐causing potential of the p.(Pro1211Arg) mutation by introducing it into recombinantly produced human CPS1. The mutant protein was expressed similarly to wild‐type CPS1 in the insect cell system used (Figure 2B, tracks under “Initial extract”), but a smaller fraction of the CPS1 produced was soluble (tracks under “Supernatant”) for the mutant than for wild‐type CPS1, suggesting that this mutation favors CPS1 misfolding. Nevertheless, the mutant form could be purified in soluble form (Figure 2C, gel picture) although in lower yield than wild‐type CPS1 (Figure 2C, first line under the gel), and it was enzymatically active, although it exhibited only ~50% of the specific activity exhibited by the pure wild‐type enzyme (Figure 2C, bottom row under the gel). Kinetic assays for all the substrates and for NAG (illustrated for ATP in Figure 2D) showed that the decreased activity was largely due to reduction in the *V*_max_ of the enzyme (Figure 2D,E, top panel). Nevertheless, the mutation increased ~5‐fold the apparent *K*_m_ for ATP (ie, it decreased the apparent affinity for ATP; Figure 2D) without inducing substantial negative changes in *K*_m_ values for bicarbonate and ammonia or for NAG (Figure 2E, bottom panel). In fact, it induced a relatively modest but significative decrease in the *K*_a_ for NAG, illustrating the fact that NCG can be efficacious in CPS1D even when the CPS1 NAG site and affinity for the activator are not directly hampered. The mutation also reduced the thermal stability of CPS1 (~4°C decrease in the temperature required for 50% inactivation) when compared with that of wild‐type CPS1 (Figure 2F), and thus it destabilized CPS1.

## 4 DISCUSSION

Given its lack of significant toxicity,^24^ NCG may be given orally in an emergency setting of undiagnosed first hyperammonemic episode, as in our patient.^2^ Indeed, it has been used in single cases of ornithine transcarbamylase deficiency^25^ and citrullinemia type 1.^26^ Observations in CPS1D indicated that the deficiency must be partial to be NCG‐sensitive,^11^, ^12^ and suggested that the specific mutations found in each patient can determine the degree of response to NCG.^12^, ^13^ The homozygous p.(Pro1211Arg) novel mutation found in our patient is shown here to cause partial deficiency and was associated with partial susceptibility to NCG. Although no responsiveness to NCG would have been observed if the patient would have hosted biallelic null mutations (such as a frameshift or premature stop mutations, which characteristically give early onset presentation), this case importantly highlights that a neonatal presentation is not evidence of null‐CPS1 activity and that an NCG trial is warranted in patients regardless of age of presentation. Of course, if the genetic diagnosis is known, the patient should carry at least one allele with a missense mutation. In any case, the responsiveness to NCG in CPS1D, as exemplified in our patient, appears much less complete than that expected in patients with NAGSD, a condition fully responsive to NCG, allowing a normal diet without any protein restriction and eliminating the need for liver transplantation.^2^

**Figure 3:**
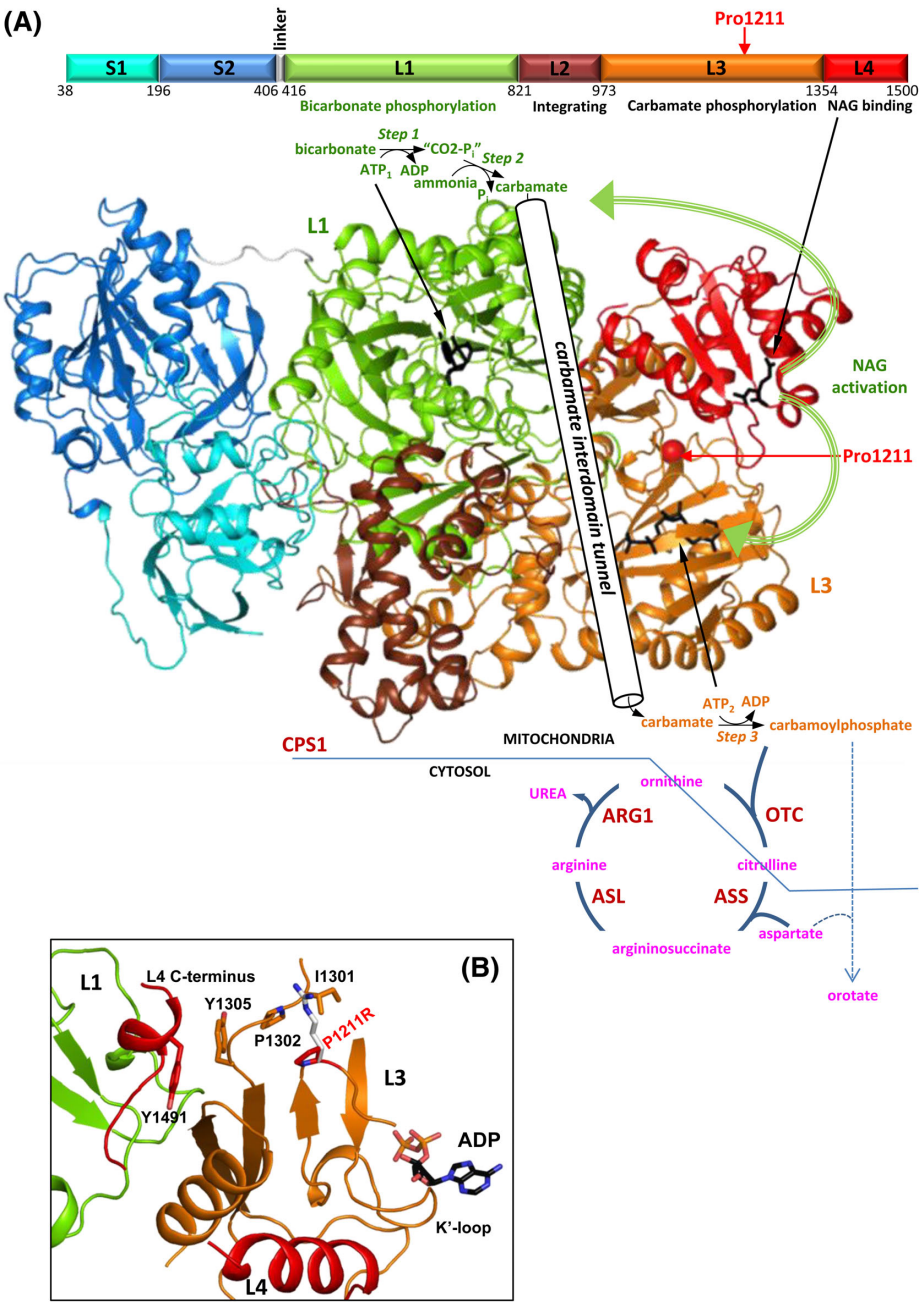
Human CPS1, its role in the urea cycle, and the CPS1D‐causing mutation p.(Pro1211Arg). Representations of 3‐D structures correspond to the Protein DataBank (PDB; http://www.rcsb.org/pdb/) file 5DOU for NAG‐activated, adenosine‐5′‐diphosphate (ADP)‐bound human CPS1.^5^ A, *Top*, linear scheme of the mature CPS1 polypeptide with the different domains highlighted in various colors and named as in De Cima et al.,^5^ giving the amino acid numbering at domain boundaries, and the functions of the domains (when known). The position of the p.Pro1211Arg mutation is marked with a red arrow. *Large structure*, experimental structure of human *CPS1* shown in cartoon representation with the domains colored as in the linear scheme. The two nucleotide molecules involved in the reaction are shown in black backbone representation, identifying their roles in the CPS1 reaction with arrows. This reaction, with its three steps, is schematized on top and bottom of the large structure, and the carbamate tunnel that connects both phosphorylation sites has been schematized nonrealistically to symbolize the intramolecular migration of the carbamate from one site to the other. The activator NAG is also represented in black sticks and marked with an arrow. The thick curved green arrows illustrate the fact that NAG activates CPS1 by influencing both phosphorylation sites and by determining the formation of a well‐shaped carbamate tunnel. Pro1211 has been marked with a red sphere and an arrow. The carbamoyl phosphate emerging from the enzyme has been integrated into a highly schematic view of the urea cycle, where classical enzymes and intermediates are labeled (OTC, ASS, ASL, and ARG1 denote ornithine transcarbamylase, argininosuccinate synthetase, argininosuccinate lyase, and arginase 1, respectively), with compartmentation between mitochondria and cytosol symbolized by the blue straight lines. The fact that carbamoyl phosphate, when accumulating as in OTC deficiency results in outflow from the mitochondria and in excessive feeding of the pyrimidine biosynthesis pathway resulting in increased urinary orotate excretion has been schematized too. B, Close up on the site where Pro1211 is located to show that it is at the cross‐roads of both phosphorylation domains and the NAG‐binding domain (each domain in a color and labeled as L1, L3, and L4, respectively), not far from the site for the ATP molecule (ADP in the crystal structure) used in the final phosphorylation step that yields the carbamoyl phosphate, and also close to the very important for activity C‐terminal extension (L4 C‐terminus). The nearby catalytic K′ loop is also labeled. The replacement of Pro1211 (red) by Arg (white) should cause a clash with Ile1301 and Pro1302, likely introducing structural instability and a suboptimal active conformation. Individual amino acids and ADP are illustrated in sticks representation and are labeled using single‐letter code. CPS1, carbamoyl phosphate synthetase 1; CPS1D, CPS1 deficiency; NAG, *N*‐acetyl‐l‐glutamate

The p.(Pro1211Arg) mutation is shown here to disfavor proper CPS1 folding, also causing protein destabilization and negatively affecting intrinsic catalysis parameters, lowering the *V*_max_ and increasing the *K*_m_^ATP^. Pro1211 maps in the carbamate phosphorylation domain of this multidomain protein that catalyzes a three‐step reaction, with one active site for each phosphorylation step, and with both sites connected by a long intervening tunnel (Figure 3A).^5^ The drastic Pro>Arg change replaces an apolar, fold‐restricting imino acid by the large and strongly basic arginine, expectedly causing some structural change at a site that is at the cross‐roads between both phosphorylation domains, the NAG‐binding domain and the C‐terminal extension that lies at the junction of both catalytic domains (Figure 3B). Thus, structural changes therein have the potential to influence overall enzyme folding, stability, and the important role of the C‐terminal extension^5^ in preventing CPS1 deactivation in each catalytic cycle. By hampering this last function, the Pro1211Arg substitution might cause the observed reduction in *V*_max_. In addition, Pro1211 is at the end of the K′ loop that is crucial for binding ATP (Figure 3B) so displacement of this loop by the structural derangement due to the substitution of proline1211 by arginine could distort somewhat this ATP site, explaining the increased *K*_m_^ATP^.

The apparent positive effect of NCG in our patient could stem from the selection for a well‐folded stable CPS1 conformation by the simultaneous binding of NCG and MgATP to the enzyme. This previously reported chaperoning effect^8^ could coexist with a kinetic effect due to prevention of subsaturation of the NAG site, as such subsaturation increases the apparent *K*_m_^ATP^ of CPS1,^27^ which in our patient would result in an unsurmountably high operative *K*_m_^ATP^. A subsaturation effect due to decreased affinity for NAG could explain the NCG sensitivity of the p.(Arg850Cys) mutation found in subject 5 of the previous 3‐day trial of NCG in CPS1D,^12^ as this mutation increased nearly 20‐fold the *K*_a_^NAG^.^28^ As illustrated here for the p.(Pro1211Arg) mutation, those mutations that increase the tendency of CPS1 to misfold and/or that decrease CPS1 stability also are candidates to being sensitive to potential chaperoning effects of NCG. This may be the case for the second CPS1D allele found in subject 5 and for the mutation found in subject 2 of the above mentioned ureagenesis augmentation trial,^12^ p.(Thr961Ile) and p.(Ala949Thr), respectively. Both mutations map in the integrating domain of CPS1 (Figure 3A), where mutations tend to cause misfolding and/or decreased stability.^28^ Indeed, the p.(Ala949Thr) mutant exhibited importantly decreased thermal stability.^28^ Chaperoning effects might also explain the NCG‐responsiveness of the CPS1D patient reported by Williams et al.,^11^ in whom both mutations [p.(His243Pro) and loss of amino acids 208‐237] mapped in the glutaminase‐like domain that, although catalytically inactive (Figure 3A), may have very important effects on enzyme folding and stabilization.^5^, ^29^ Thus, a corollary of all the above is that potential chaperoning effects of NCG should be kept in mind because they may be crucial in determining NCG effectiveness in CPS1D. As mutation effects on CPS1 stability may not be easily predictable, and given the poor general prognosis of CPS1D, it appears reasonable to give patients a clinical trial of NCG to assess the clinical responsiveness to this orphan drug, taking due caution with monitoring for potential negative effects of NCG as observed in a single CPS1D patient.^13^

## COMPLIANCE WITH ETHICS GUIDELINES

This is an observational retrospective patient study that did not involve any human experimentation or research‐based patient intervention, as all interventions and decisions made were intended to diagnose and treat the patient. In any case, no aspect of the present report is in contradiction with the Helsinki Declaration of 1975, as revised in 2000.

## CONFLICT OF INTEREST

Sufin Yap has received honoraria, travel and accommodation support from Orphan Europe‐Recordati Rare Disease for participation in industry‐sponsored symposia, advisory board meetings, and master classes and attending scientific meetings. Nadine Gougeard, Antony R. Hart, and Belén Barcelona declare that they have no conflict of interest. Vicente Rubio has received honoraria, travel and accommodation from Orphan Europe‐Recordati Rare Disease for participation in company‐sponsored symposia, and advisory board meetings.

## INFORMED CONSENT

Written informed consent for the publication of these data was obtained from the patient's mother.

## ANIMAL RIGHTS

This article does not contain any studies with animal subjects performed by any of the authors.

6

## AUTHORS' CONTRIBUTIONS

S.Y. diagnosed and treated the patient, conceived the study, obtained consent, provided the clinical data and Figure 1 and, jointly with V.R., wrote the paper with initial help from an external editorial assistant. A.H. gave neurological input and performed the neurodevelopmental assessment. N.G. and B.B. carried out the experimental studies under V.R. guidance, interpreted the experimental results on the light of the CPS1 structure, and prepared Figures 2 and 3. All authors assisted in the revision of the draft and approved it before submission, including the author's order. S.Y. serves as guarantor for the article, accepts full responsibility for the work and the conduct of the study, had access to the data, and controlled the decision to publish.

## Data Availability

Primers' sequences used in this study will be provided on request. All other information is provided in the manuscript.
